# Effect of Silver Annealing Conditions on the Performance of Electrolytic Silver/Silver Chloride Electrodes used in Harned Cell Measurements of pH

**DOI:** 10.3390/s100302202

**Published:** 2010-03-17

**Authors:** Paul. J. Brewer, Richard. J. C. Brown

**Affiliations:** Analytical Science Division, National Physical Laboratory, Teddington, Middlesex TW11 0LW, UK; E-Mail: richard.brown@npl.co.uk

**Keywords:** pH, Harned cell, Ag/AgCl electrodes, annealing, stability

## Abstract

We have studied the long and short term stability of electrolytic Ag/AgCl electrodes fabricated from Ag wire that has been subjected to a range of different annealing conditions. At elevated temperatures, the presence of oxygen during the annealing process has been shown to be detrimental to the performance of electrodes produced. This phenomenon has been attributed to the dissolution of oxygen in the Ag lattice leading to structural changes in the Ag/AgCl electrode material. Electrodes prepared from Ag wire annealed in the absence of oxygen have shown no appreciable change in performance throughout the temperature range employed. This work has resulted in an improved understanding of the optimum annealing conditions required for Ag used in the preparation of electrolytic Ag/AgCl reference electrodes. This work has positive implications for the accuracy of Harned cell measurements of pH.

## 1. Introduction

Sørensen introduced the concept of pH in 1909 [1]. Since then, measurements of pH have become increasingly more widespread, impacting on a vast number of market sectors [2–5]. Examples of areas where pH measurements are frequently made are blood gas determinations, chemical and drug manufacture, food and drink processing, water purity, and effluent discharge control. Due to the significant economic and societal consequences of measurement inaccuracies it is essential to ensure validity and traceability [6,7].

An electrochemical cell arrangement referred to as the “Harned cell” is used to determine primary pH standard values. The cell is unique in that it does not contain a liquid junction and relies on well characterised Ag/AgCl reference electrodes for operation [8]. It has the potential to be a primary method for the absolute measurement of pH, providing that it can conform to the accepted definition of a primary method [9] that requires a methodology and operation that can be completely described and understood, for which a complete uncertainty statement can be written down in terms of SI units.

Usually, in metrological applications, excluding the determination of the molality of HCl used in the Harned cell, the reference potential of Ag/AgCl electrodes employed is the largest contribution to the measurement uncertainty of pH [8]. The stability of the potential when Ag/AgCl electrodes are transferred between different electrolytes was first commented upon by R. G. Bates in the 1940s and has since been re-investigated by K. W. Pratt [10]. Transfer of an electrode between solutions induces a large initial change in the reference potential (as compared to a Ag/AgCl reference electrode which has been allowed enough time to reach equilibrium in a new solution). This initial change then decays as the Ag/AgCl reference electrode reaches an equilibrium potential in the new solution environment. These shifts in electrode potential can have significant implications for the accurate operation of the Harned cell and the throughput of the certification of primary reference buffers. Hence, improving the long and short term stability of Ag/AgCl reference electrodes is crucial in the quest to drive down the uncertainty of primary pH measurement.

Several methodologies have been developed for preparation of Ag/AgCl electrodes and, amongst these, two remain widely employed. The first, known as the ‘electrolytic type’ is prepared by electrolytically converting a fraction of pure Ag to AgCl. The structure of this material was first investigated by Huber with electron microscopy [11] and has since been reviewed and reported by Janz *et al.* [12]. Electron microscopy revealed a morphology with little porosity that was later referred to as a ‘locked’ structure. The second method is by ‘thermal electrolytic’ means. The electrode consists of a sphere of Ag/AgCl on a Pt wire and is prepared from a porous Ag_2_O paste by a two stage process. The material is first heated to produce Ag and then electrolytically converted to produce Ag/AgCl. In contrast to the locked structure, Janz proposed two alternative structures for this material both containing a high degree of porosity. The highly porous structure was later confirmed by Brown *et al.* [13] following an investigation of the timescale of the equilibrium process when a thermal electrolytic Ag/AgCl electrode is transferred for one solution to another. They reported the effect of the diameter of the Ag/AgCl sphere used in the electrodes on the equilibration time and concluded the presence of a porous structure that limits the rate at which traces of any previous solutions are diluted by any new environment. Larger diameter spheres of Ag/AgCl were shown to require longer times to reach equilibrium which is consistent with the process being described by diffusion whereby traces of the previous solution diffuses out of the pore structure while the new solution diffuses in. In a previous study [14], the long term stability of thermal electrolytic Ag/AgCl electrodes was shown to be superior to the electrolytic type as a result of the higher geometric surface area and exchange current density (the equilibrium rate of oxidation or reduction at an electrode expressed in terms of current) present in the porous structure. Owing to their enhanced long term stability [15] and better repeatability of the manufacturing process, thermal electrolytic electrodes have become the conventional choice for use in the Harned cell. Hence electrodes of this type have been studied extensively to optimise performance to meet the requirements for high quality results [16].

Despite their limitations, electrolytic Ag/AgCl electrodes offer several advantages over the thermal electrolytic type. As well as a less time-consuming fabrication procedure, the low degree of porosity in the Ag/AgCl structure results in substantially reduced equilibration times after transfer to a new solution compared to the porous thermal electrolytic type. This ability to adapt to a new solution environment on a short time scale is crucial for making accurate Harned cell measurements as it increases the probability that the electrodes will be at equilibrium within the typical timescales employed and reduces the risk of contaminating the solution to be measured by transference of a previous solution (as a result of transfer in the porous structure). Due to the fragile nature and storage requirements of thermal electrolytic electrodes, a major drawback is their lack of portability. Electrolytic electrodes are more robust as a result of being manufactured from Ag wire as opposed to porous Ag (produced from heating Ag_2_O paste). Hence a further advantage of the electrolytic type is their potential for use as a portable sensor as they can be transported with less rigorous packing. Hence if the long term stability and repeatability of electrolytic Ag/AgCl electrodes are improved, these advantages can be exploited.

Silver is used in a variety of applications such as electronics due to the requirement for high conductivity that it possesses even when tarnished. Prior to use, the material is annealed in order to increase its ductility in preparation for work such as shaping, stamping and forming. Annealing is carried out by heating Ag (often until the material is glowing) for a fixed time period. The Ag is then allowed to cool slowly. Annealing occurs by diffusion of atoms that progresses a material towards its thermodynamic equilibrium state. Heat increases the rate of diffusion by providing the energy required to break bonds. The movement of atoms has the effect of redistributing and destroying the crystallographic defects allowing Ag to deform more easily and produce an homogenous material. Hence annealing is likely to remove strains in Ag wire introduced by the drawing process and improve structural uniformity which may lead to an increase in performance of electrolytic Ag/AgCl electrodes. In this paper, Ag wire has been subjected to different annealing conditions. Electrolytic Ag/AgCl electrodes have been made from the Ag wire in order to study the effect of annealing on the long and short term stability and repeatability of electrodes. This work has resulted in an improved understanding of the optimum annealing conditions for electrode fabrication and should have positive implications for the accurate operation of the Harned cell.

## 2. Results and Discussion

**Figure 1.**
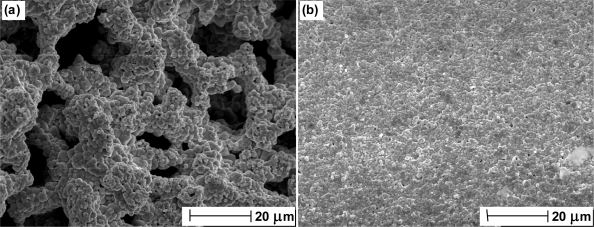
SEM images of (a) the spherical Ag/AgCl material from a thermal electrolytic electrode and (b) the Ag/AgCl material from an electrolytic electrode.

We have used Scanning Electron Microscopy (SEM) to confirm the structure of thermal electrolytic and electrolytic Ag/AgCl electrodes. Figure 1 (a) is an SEM image from the Ag/AgCl sphere of a thermal electrolytic electrode. The structure shows a high degree of porosity. In contrast, the SEM image of the Ag/AgCl material from an electrolytic electrode shown in Figure 1 (b) reveals very little or no pore structure.

**Figure 2.**
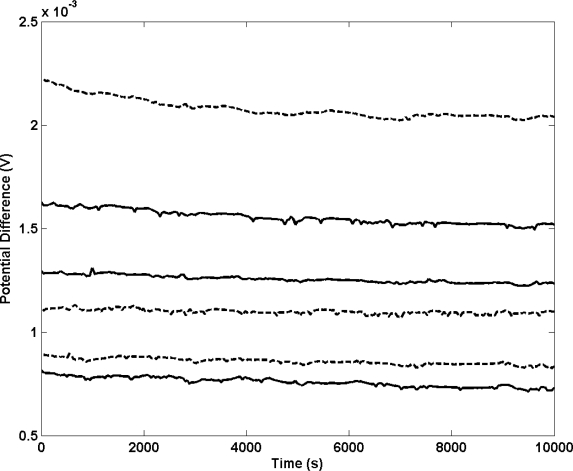
Transient potential difference measurements for electrolytic Ag/AgCl electrodes equilibrated in a 0.01 M HCl solution *vs.* a thermal electrolytic defacto reference electrode. Each profile corresponds to an individual electrode. Electrodes were prepared using Ag wire that had no pretreatment (solid lines) and a bending process whereby the material was flexed ten times along the 0.5 cm length (dashed lines).

Figure 2 shows the differential potential transients of a selection of electrolytic Ag/AgCl electrodes compared to a thermal electrolytic Ag/AgCl defacto reference. Electrodes manufactured with no pretreatment of the Ag material prior to electrolytic conversion to Ag/AgCl are represented with solid lines. The long term stability of these electrodes display the typical behaviour shown in previous work [14] and deviate in each case by about 100 μV across the measurement period of approximately 3 hours (chosen to represent the time period typically employed for making Harned cell measurements of pH). As expected, the transients are poorly repeatable and would not meet the specification for use in the Harned cell as the bias between any two electrodes is large (with the largest difference being approximately 800 μV). A large bias in potential between these electrodes and the thermal electrolytic defacto reference also exists (approximately 1 mV) as has previously been reported [14] and attributed to strains and probable impurities introduced in the drawing process of the Ag wire used to produce the electrodes. This effect might also account for the variability in potential between electrodes [17]. An experiment was conducted to investigate whether the repeatability of the potential transient and the bias with respect to the defacto reference would worsen if further strains were introduced to the wire. Electrolytic Ag/AgCl electrodes were prepared using Ag wire that had undergone a bending process whereby the material was flexed in a controlled manner (*i.e*., backwards and forwards ten times with a bend radius of 2.5 mm). The resultant ensemble of potential transients (dashed lines) display no appreciable differences to the transients from electrodes manufactured with no pretreatment of the Ag wire. This suggests that the bias in potential between electrodes and the defacto reference cannot be altered significantly from poor handling and is most likely attributed to the variability in the drawing process of the wire from strains or introduced contaminants.

**Figure 3.**
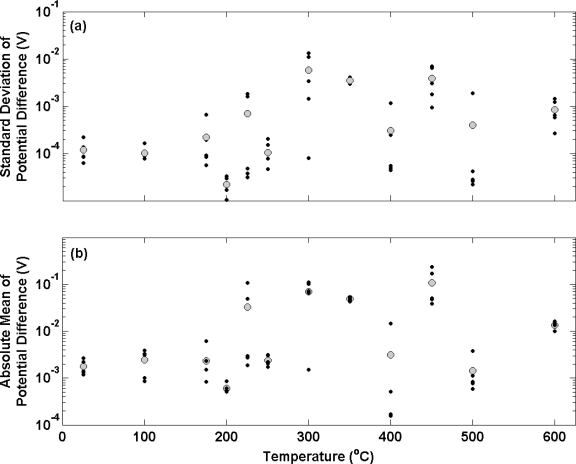
(a) Standard deviation and (b) absolute mean of the potential difference *vs.* a thermal electrolytic defacto reference electrode for electrolytic electrodes equilibrated in a 0.01 M HCl solution plotted as a function of annealing temperature in air. The smaller black circles correspond to the standard deviation (a) and mean (b) of an individual electrode measured over a time period of 3 hours respectively. The mean of the ensemble at each temperature in both sub-plots is shown by the larger grey circles.

Figure 3 (a) shows the standard deviation of potential difference measurements over an approximate 3 hour period between a series of electrolytic Ag/AgCl electrodes and a thermal electrolytic defacto Ag/AgCl reference electrode equilibrated in a 0.01 M HCl solution. Ag wires used to make the electrolytic electrodes were subjected to different annealing temperatures in air (covering the range 20–600 °C) for 2 hours prior to anodisation. Black and grey circles represent the standard deviation of an individual electrode and the mean standard deviation of the ensemble at each temperature respectively. The standard deviation of the differential potential transient for each electrode has been used as a measure of long term stability. The figure shows that electrodes manufactured from Ag annealed at temperatures of 100 °C or lower, display reasonably good long term stability with a mean standard deviation for the ensemble of approximately 100 μV. When the temperature of the annealing process is elevated to above 100 °C, the long term stability of electrodes becomes more variable. In some cases the long term stability is improved (for example some electrodes produced from Ag annealed at 200 °C, 400 °C and 500 °C produce standard deviations of less than 100 μV). The majority of electrodes however exhibit poorer long term stability when compared to those produced from Ag annealed at temperatures of 100 °C or lower. Figure 3 (b) shows the absolute mean (to enable a log scale) of the potential transient for each electrode over the time period of approximately 3 hours (represented with black circles). The mean of the ensemble at each temperature is shown by the larger grey circles. This graph displays the mean bias between each electrode and the defacto reference. The electrodes prepared with Ag annealed at temperatures of 100 °C or less exhibit a mean bias of about 1 mV to the defacto reference that is consistent with the measurements in Figure 2. Both electrode ensembles exhibit reasonable repeatability with the largest bias from the mean of each ensemble being no greater than 1.5 mV. In accordance with Figure 3 (a), electrodes prepared from Ag annealed at temperatures greater than 100 °C display poorer repeatability. The bias between individual electrodes produced from Ag annealed at higher temperatures is considerably larger with the greatest bias from the mean in an ensemble being 100 mV (electrodes prepared from Ag wire annealed at 450 °C).

**Figure 4.**
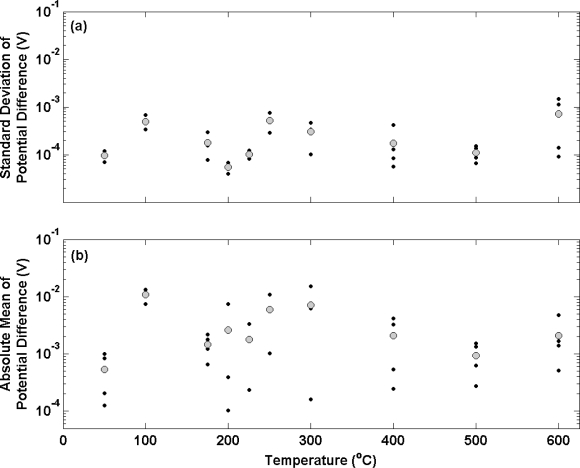
(a) Standard deviation and (b) absolute mean of the potential difference *vs.* a thermal electrolytic defacto reference electrode for electrolytic electrodes equilibrated in a 0.01 M HCl solution plotted as a function of anneaing temperature in nitrogen. The smaller black circles correspond to the standard deviation (a) and mean (b) of an individual electrode measured over a time period of 3 hours. The mean of the ensemble at each temperature in both sub-plots is shown by the larger grey circles.

Figure 4 shows the results of potential transient measurements for a second batch of electrolytic Ag/AgCl electrodes following the same data analysis used in figure 3. In this experiment, electrodes were prepared from Ag wire that had been subjected to the same annealing temperatures as before (covering the range 20–600 °C) for 2 hours. In this case, annealing was performed in a nitrogen environment in order to investigate the effect on oxygen on electrode stability. Electrodes prepared from Ag annealed at temperatures at 100 °C or below display similar characteristics to electrodes prepared at equivalent annealing temperatures in air (Figure 3). The mean standard deviation for the two electrode ensembles prepared with annealing temperatures up to 100 °C in Figure 4 (a) is approximately 100 μV (in accordance with the data in figure 3 (a)). These electrodes also display a similar bias (about 1 mV) to the defacto reference compared to the electrodes at equivalent annealing temperatures in Figure 3 (b). When annealing temperatures in nitrogen were increased, instead of displaying poorer stability and less repeatable potential differences (figure 3), electrodes show no appreciable change in performance. The standard deviation of each individual electrode over the temperature range deviates very little from 100 μV with the mean of any ensemble not exceeding 1 mV. Figure 4 (b) presents the bias of individual electrodes from the thermal electrolytic defacto reference. In contrast to Figure 3 (b), the Ag annealing temperature (in the absence of oxygen) does not appear to influence the repeatability of the electrode potential difference measurements. The repeatability is shown to be consistent throughout the range of temperatures and is a vast improvement on Figure 3 (b) with no electrode deviating by more than 1 mV from the mean of the electrode ensemble. In addition the electrodes exhibit a substantially smaller bias from the reference than those in Figure 3 (b) across the range of annealing temperatures employed.

**Figure 5.**
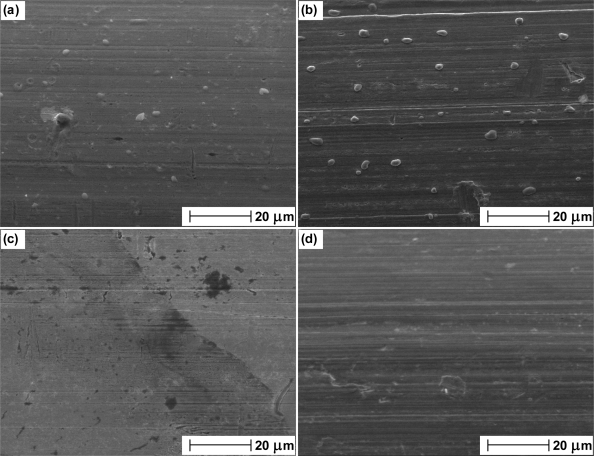
SEM images of Ag wire following a 2 hour annealing step in (a) air at 100 °C, (b) air at 400 °C, (c) nitrogen at 100 °C and (d) nitrogen at 400 °C. In each case the wire was placed in an atmosphere of hydrogen.

The presence of oxygen in the annealing process of Ag at temperatures greater than 100 °C is detrimental to the long term stability and repeatability of electrolytic Ag/AgCl electrodes. An explanation can be offered by considering the role of oxygen in the annealing process. It is known that oxygen in contact with metallic Ag dissolves into the bulk. The higher the temperature and pressure, the higher is the level of dissolved oxygen. [18]. The interaction of Ag with oxygen was studied by Rehren *et al.* using XPS and SEM [19]. At lower temperatures, Ag has a low susceptibility to oxidation due to the presence of adsorbed atomic oxygen and co-adsorbed CO_2_. Above the temperature at which this layer is desorbed, Rehren *et al.* attributed the continued low reactivity of Ag to dissolved oxygen. Measurements were supported by considering the structural conditions of the Ag lattice as atomic oxygen fits perfectly into the octahedral holes enabling dissolution without significantly changing the atomic distances of the Ag solid. Dissolved oxygen may account for the impaired long term stability and increased bias in potential difference between electrodes produced from Ag annealed in air at temperatures greater than 100 °C due to its impact on the structure and morphology of the material. Its presence in Ag annealed in air has been confirmed with the SEM images in figure 5. SEM images of Ag wire annealed in air at 100 °C and 400 °C are shown in (a) and (b) respectively. Prior to collection of SEM images, each wire was placed in an atmosphere of hydrogen. A small degree of pitting is present on the surface of Ag wire annealed at 100 °C (as a result from the reaction of dissolved oxygen with hydrogen to produce water). The degree of pitting is shown to increase in the sample annealed at 400 °C (Figure 5 (b)). These micrographs (that are representative of the whole area) show that Ag is an effective reservoir for dissolved oxygen, with the uptake increasing at higher temperatures. The data in figure 3 indicates a threshold at which the amount of dissolved oxygen influences the Ag/AgCl material as above 100 °C the impaired stability does not appear to increase with temperature. Although the image at 100 °C reveals the presence of some dissolved oxygen, the results in Figure 3 at this temperature indicate that it is not enough to cause noticeable affects on electrode stability. In contrast SEM images of Ag wire annealed in nitrogen at 100 °C and 400 °C (Figures 5 (c) and 5 (d) respectively) present almost featureless images of structures with no pitting indicative that oxygen has not dissolved. Faint horizontal lines are present in the micrographs from all samples and can be attributed to the drawing process.

**Figure 6.**
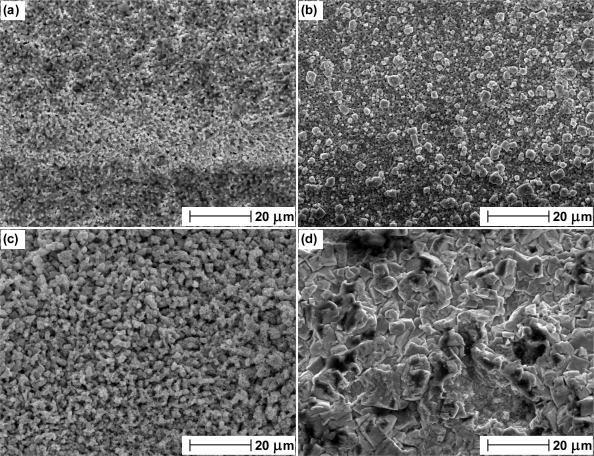
SEM images of Ag/AgCl produced by anodisation of Ag wire following a 2 hour annealing step in (a) air at 100 °C, (b) air at 400 °C, (c) nitrogen at 100 °C and (d) nitrogen at 400 °C.

Figure 6 presents SEM images of the Ag wires in Figure 5 after anodisation to Ag/AgCl (used in the electrode manufacturing process). Figures 6 (a) and (b) present the microstructure of the Ag/AgCl material produced from Ag annealed at 100 °C and 400 °C in air respectively. As expected the image in (a) reveals a structure with little porosity (in accordance with Figure 1 (b)). The morphology of the material in Figure 6 (b) somewhat differs to (a) with the presence of larger grains on the surface. In addition the porosity of the material in Figure 6 (b) appears to have been reduced with fewer darker areas indicative of craters in the solid. Hence the images suggest that the presence of dissolved oxygen alters the porosity and surface of the material and explains the impaired long term stability due to a lower exchange current density (resulting from a reduced active area) available to define the equilibrium voltage and any continued movement of oxygen within the lattice. Hence any changes in electrode performance due to dissolved oxygen are likely to be attributed to physical changes in Ag/AgCl structure induced from the presence of oxygen in the anodisation process. Figures 6 (c) and (d) are SEM images taken from the Ag/AgCl material produced from Ag annealed at 100 °C and 400 °C in a nitrogen environment and show changes in morphology that are not present in Figure 5. Figure 6 (c) presents a vastly different structure to (a) and (b) with the appearance of larger crystals and more pore structure. Figure 6 (d) is even more remarkable as the Ag/AgCl microstructure is shown to have developed into a “ribbon” like structure as a result of annealing at the higher temperature. Although the scale length for features is larger, the morphology appears to be quite smooth, hence it would be expected that the exchange current density and long term stability is similar to electrodes produced at lower temperatures in nitrogen. This is supported from the long term stability studies in figure 4 as electrodes produced at lower temperatures display similar stability characteristics to those prepared from Ag annealed at higher temperatures.

**Figure 7.**
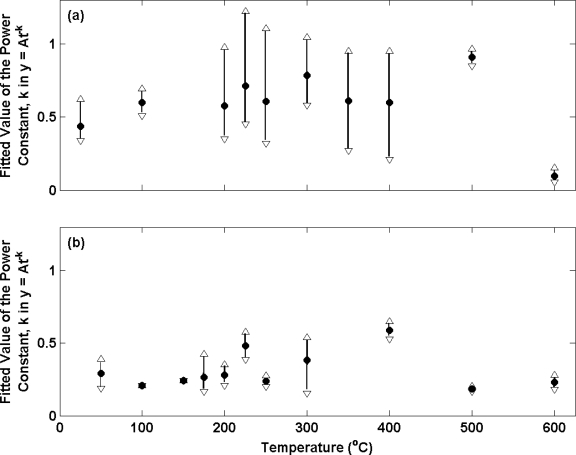
Transient potential difference measurements for electrolytic Ag/AgCl electrodes *vs.* a thermal electrolytic defacto reference electrode after transfer from a 0.01 M HCl solution to a 0.025 M Na_2_HPO_4_/ 0.025 M KH_2_PO_4_ buffer solution. The electrodes were equilibrated in a 0.01 M HCl solution prior to transference. The value of the exponent, k, in the power function of type y = A t^−k^ was fitted to the first 60 s of the transient potential difference measurements and is plotted as a function of annealing temperature for Ag wire annealed in (a) air and (b) nitrogen. The black circles represent the mean power constant determined for the ensemble of electrodes at each annealing temperature. The triangles indicate the upper and lower power constants determined for the electrode ensemble at each temperature.

Figure 7 presents the potential transients for the electrolytic Ag/AgCl electrodes used in measurements presented in figure 3 and 4 after transfer from a 0.01 M HCl solution to a 0.025 M Na_2_HPO_4_/ 0.025 M KH_2_PO_4_ buffer solution. Electrodes prepared from Ag annealed in air and nitrogen are shown in (a) and (b) respectively. The data has been normalised to the voltage at t = 0 for each transient. For each electrode the potential differences are initially large and decay with time. The observed decay with time for the first 60 s (the time period in which a significant rate of change of potential is observed) has been fitted to a power function of the type (y = At^−k^) where t is the time elapsed in seconds, y is the normalised voltage difference and A is the normalised voltage difference when t = 0 (as carried out in previous work [14]). Only data for the first 60s has been considered here as the majority of the progress towards achieving equilibrium occurs in this time period hence making comparisons between electrodes clearer than with the data after 60 s. A power function was selected as this provided the best fit to the data. At each annealing temperature the mean (filled circles), maximum (open upward triangles) and minimum (open downward triangles) exponents for the electrode ensemble at each temperature is presented.

The results in (a) are in accordance with the long term stability measurements in Figure 3 as they display more variable behaviour (larger biases between the maximum and minimum in each electrode ensemble) for electrodes produced from Ag annealed in air at temperatures in excess of 100 °C. For example, the electrode with the largest exponent at 250 °C has a bias of 0.5 from the mean of the ensemble, compared to the electrode with the largest exponent at 100 °C (bias of 0.1 to the mean of the ensemble). Measurements at 500 °C and 600 °C are an exception and display data sets with little dispersion. However, the mean of these ensembles is significantly biased from the data measured at the other annealing temperatures. In contrast, the repeatability of electrode performance is shown to vastly improve upon changing from air to nitrogen for annealing across the entire range of annealing temperatures (figure 7 (b)). This is in accordance with figure 4. For example, the electrode with the largest exponent at 250 °C now has a bias of only 0.04 from the mean of the ensemble.

It is interesting to note that the mean of all electrodes in (b) is somewhat smaller than the same from (a) (0.31 compared to 0.59 respectively). This parameter is a measure of the speed at which an electrode achieves equilibrium in a new solution and suggest that on average electrodes prepared from Ag annealed in air are faster to respond. This observation is consistent with the structures presented in figure 6. Electrodes manufactured from Ag annealed in nitrogen were shown to contain a slightly more porous structure than those produced from Ag annealed in air. The porosity is likely to limit the rate at which the electrodes can equilibrate, as any previous solution contained within the pores of the Ag/AgCl must diffuse out before a stable potential can be achieved (similar to previous observations [13]).

## 3. Experimental Section

Electrolytic Ag/AgCl reference electrodes were prepared following the standard procedure outlined in the literature [20–22]. This involved anodisation of Ag wires (0.5 mm diameter, 5mm length) to convert approximately 15% to AgCl. A constant current density of 1 mA/cm^2^ of Ag was passed for 1 hour in a 0.1 M HCl solution. Prior to anodisation Ag wires annealed in a tube furnace for 2 hours at a variety of different temperatures ranging from 20 to 600 °C. The annealing process was conducted with 2 different environments: air and N_2_ (Air Products, BIP grade). After cooling, each Ag wire was immersed in a 0.1 M HCl solution to remove surface contaminants and then rinsed thoroughly with distilled water.

A defacto Ag/AgCl reference electrode was prepared following the thermal electrolytic method [20–23]. This electrode type is the most widely used in Harned cell measurements and provides good long term stability. A set of 4 electrodes were prepared by thermal decomposition (100 °C for 1 hour followed by 500 °C for 2 hours) of three separate applications of Ag_2_O paste to a Pt wire (99.99%, 0.5 mm diameter). Following this process, approximately 15% of the material was electrolytically converted to AgCl in a solution of 0.1 M HCl. The amount of Ag on each electrode was determined prior to anodisation and the charge required to convert 15% to AgCl was calculated. The appropriate current was then applied to achieve this charge over a 1 hour period. The long term stability of the 4 electrodes was tested by simultaneously measuring the potential difference of each electrode against a fifth thermal electrolytic Ag/AgCl electrode (previously manufactured and conditioned) for a period of 24 hours. An electrode with a potential that differed by less than 100 μV from the mean of the ensemble and with a standard deviation of less than 100 μV (over a period of 3 hours) was selected as the defacto reference for the work in this paper.

Measurements to investigate the long term stability of the Ag/AgCl reference electrodes were carried out by placing the defacto thermal electrolytic reference in a 0.01 M HCl solution with the electrolytic Ag/AgCl electrodes under test and allowing a period of at least twelve hours for equilibrium to be reached. All of the potentials are reported with respect to the defacto electrode. In a separate experiment to test the timescale of the equilibration process, the defacto reference was placed in a solution of 0.025 M Na_2_HPO_4_/0.025 M KH_2_PO_4_ buffer and allowed to equilibrate for at least twelve hours. The electrodes under observation were then removed from the 0.01 M HCl solution and transferred to the phosphate buffer solution ensuring that any excess solution was removed. In both experiments, the potential difference between the electrodes and the reference was measured as a function of time using a high accuracy 18-Bit National Instruments multifunction data acquisition device (USB-6289). Measurements were acquired using software written in LabVIEW 8.

All solutions were prepared using 18.2 MΩ cm distilled and deionised water (MilliQ, Millipore) and ultra high purity chemicals (Fisher, UK). All experiments were carried out with solution temperatures of 20 ± 2 °C. All solutions were degassed with nitrogen before use (metrology grade, BOC UK). A positive pressure of nitrogen was maintained over the solution during experimentation.

To study the structure of Ag used to prepare Ag/AgCl electrodes and the anodized Ag/AgCl material, secondary electron images were collected using a Camscan MX2500 scanning electron microscope. An accelerating voltage of 20 kV was used for imaging.

## 4. Conclusions

We have studied the long and short term stability of electrolytic Ag/AgCl electrodes fabricated from Ag wire that has been annealed under different atmospheric conditions. In most cases, electrodes produced from Ag wire annealed in air at temperatures exceeding 100 °C were shown to exhibit impaired long term stability and less repeatable absolute potential difference measurements to a stable thermal electrolytic defacto reference. When annealing temperatures in nitrogen were increased, instead of displaying poorer stability and less repeatable potential differences as shown in the presence of oxygen, electrodes displayed no appreciable change in performance throughout the temperature range employed. Hence the presence of oxygen in the annealing process has been shown to be detrimental to the performance characteristics of electrolytic Ag/AgCl electrodes. In addition to preventing the dissolution of oxygen into Ag, the role of nitrogen in the annealing process may also be to expel any pre-existing oxygen in the Ag. SEM of Ag wires annealed in air has revealed the presence of dissolved oxygen in the Ag lattice which increases with annealing temperature. Further SEM images of Ag/AgCl material produced from electrolytic conversion of the annealed Ag suggest a change in the structural characteristics when dissolved oxygen is present. Dissolved oxygen appears to result in a structure with reduced porosity and lower geometric surface area. This structural change however is small and hence only partially explains the compromised stability of electrodes made from Ag annealed in the presence of oxygen. Therefore it is likely that the presence of dissolved oxygen during the anodisation process may cause chemical changes in the Ag/AgCl material electrode that results in the poor stability displayed, although further work is required to investigate this. Measurements of the short term stability characteristics (after transfer to a new solution environment) show electrodes produced from Ag annealed at temperatures in air in excess of 100 °C to display poor repeatability. In contrast, the repeatability of electrodes prepared from Ag annealed in nitrogen is shown to improve by an order of magnitude across the entire range of Ag annealing temperatures. However, electrodes prepared from Ag annealed in air exhibited on average, faster equilibration times after transfer to a new solution compared to electrodes produced from Ag annealed in nitrogen (mean exponent of 0.59 compared to 0.31 respectively). This observation is consistent with the deductions from SEM as electrodes manufactured from Ag annealed in nitrogen were shown to contain a more porous structure than those produced from Ag annealed in air. The porosity is likely to limit the rate at which the electrodes can equilibrate, as any previous solution contained within the pores of the Ag/AgCl must diffuse out before a stable potential can be achieved.

Minimising the bias in potential between Ag/AgCl reference electrodes is imperative for Harned Cell measurements, hence stability and repeatability of electrode potentials is of major importance. This work has resulted in an improved understanding of the optimum annealing conditions required for Ag in the preparation of electrolytic Ag/AgCl reference electrodes and the limitations for their use in Harned cell measurements of pH. Although optimised electrodes prepared from Ag annealed in nitrogen reveal an interesting structure with increased porosity, the long term stability remains inferior to thermal electrolytic electrodes. Although these optimised electrolytic Ag/AgCl electrodes are unlikely to be suitable for use in Harned cell measurements of pH, their optimised long term stability and ability to respond to a new solution environment on a short time scale gives these devices the potential for a wide range of applications such as portable sensors that can be easily manufactured in large numbers.

## References

[1] Sørensen S.P.L. (1909). Enzymstudien II. Mitteilung. Über die Messung und die Bedeutung der Wasserstoffenkonzentration bei enzymatischen Prozessen. Biochem Z.

[2] Comer J.E.A., Hibbert C.J. (1997). pH electrode performance under automated management conditions. J. Automatic Chem.

[3] Pfannenstiel E. (2002). Process pH measurement. Intech.

[4] Bates R.G. (1973). Determination of pH: theory and practice.

[5] Galster H. (1991). pH measurement.

[6] Spitzer P. (2001). Traceable measurements of pH. Accred. Qual. Assur.

[7] Brown R.J.C., Milton M.J.T. (2003). Observation of a combined dilution and salting effect in buffers under conditions of high dilution and high ionic strength. Accred. Qual. Assur.

[8] Buck R.P., Rondinini S., Covington A.K., Baucke F.G., Brett C.M., Camoes M.F., Milton M.J.T., Mussini T., Naumann R., Pratt K.W., Spitzer P., Wilson G.S. (2002). Measurement of pH. Definition, standards and procedures. Pure Appl. Chem.

[9] Comité Consultatif pour la Quantité de Matiére (CCQM)

[10] Pratt K.W. Adventures in CO3 pH(S) CRM Certification.

[11] Huber K. (1955). Microstructure of anodically formed silver halide films. Z. Electrochem.

[12] Janz J.G. (1961). Reference electrodes.

[13] Brown R.J.C., Milton M.J.T. (2005). The microporous structure of silver/silver chloride electrodes and the implications for Harned cell operation. Accred. Qual. Assur.

[14] Brewer P.J, Brown R.J.C. (2009). Effect of structural design of silver/silver chloride electrodes on stability and response time and the implications for improved accuracy in pH measurement. Sensors.

[15] Brown R.J.C., Brewer P.J., Brett D.J.L. (2009). Long-term equilibrium potential and electrochemical impedance study of Ag/AgCl electrodes used in Harned Cell measurements of pH. Accred. Qual. Assur.

[16] Guiomar Lito M.J., Camões M. (2009). Meeting the requirements of the silver/silver chloride reference electrode. J. Solution Chem.

[17] Covington A.K. (1969). Reference electrodes. Nat. Bur. Stand Spec. Publ.

[18] Brown H.C. (1971). Gmelin, Handbuch der anorganischen Chemie.

[19] Rehren C., Muhler M., Bao X., Schlögl R., Ertl G. (1991). The interaction of silver with oxygen. Z. Phys. Chem.

[20] Harned H.S. (1929). The electromotive forces of uni-univalent halides in concentrated aqueous solutions. J. Am. Chem. Soc.

[21] British Standards Institution (1979). BS 2586:1979. Specification for glass and reference electrodes for the measurement of pH.

[22] Hamer W.J., Acree S.F. (1939). Primary pH measurements and standards. J. Res. Nat. Bur. Stand.

[23] Taniguchi H., Janz G.J. (1957). Preparation and reproducibility of the thermal electrolytic silver-silver chloride electrode. J. Electrochem. Soc.

